# Prebiotic Treatment in Patients with Nonalcoholic Fatty Liver Disease (NAFLD)—A Randomized Pilot Trial

**DOI:** 10.3390/nu16111571

**Published:** 2024-05-22

**Authors:** Naama Reshef, Uri Gophna, Leah Reshef, Fred Konikoff, Gila Gabay, Taiba Zornitzki, Hilla Knobler, Yaakov Maor

**Affiliations:** 1 Institute of Diabetes and Metabolism-Kaplan Medical Center, Rehovot 7661041, Israel; taiba_z@clalit.org.il (T.Z.); hilla.knobler@gmail.com (H.K.); 2 School of Nutritional Sciences, Faculty of Agriculture, Food and Environment, The Hebrew University, Jerusalem 9112102, Israel; 3 Shmunis School of Biomedicine and Cancer Research, Faculty of Life Sciences, Tel-Aviv University, Tel Aviv 6423906, Israel; urigo@tauex.tau.ac.il (U.G.); leahfa@gmail.com (L.R.); 4 Institute of Gastroenterology and Hepatology-Meir Medical Center, Kefar Sava 4428164, Israel; konikoff@tauex.tau.ac.il (F.K.);; 5 Faculty of Medicine, Tel-Aviv University, Tel Aviv 6423906, Israel; 6 Hadassah School of Medicine, The Hebrew University, Jerusalem 9112102, Israel; halishy@netvision.net.il; 7 Institute of Gastroenterology and Hepatology-Kaplan Medical Center, Rehovot 7661043, Israel

**Keywords:** NAFLD, microbiota, prebiotic, fibroblast growth factor, lipopolysaccharide

## Abstract

Several studies show that gut microbiotas in patients with nonalcoholic fatty liver disease (NAFLD) differ from those in a healthy population, suggesting that this alteration plays a role in NAFLD pathogenesis. We investigated whether prebiotic administration affects liver fat content and/or liver-related and metabolic parameters. Patients with NAFLD and metabolic syndrome (age: 50 ± 11; 79% men) were randomized to receive either 16 g/day of prebiotic (ITFs—inulin-type fructans) (*n* = 8) or placebo (maltodextrin) (*n* = 11) for 12 weeks. Patients were instructed to maintain a stable weight throughout the study. Liver fat content (measured by H^1^MRS), fecal microbiota, and metabolic, inflammatory, and liver parameters were determined before and after intervention. Fecal samples from patients who received the prebiotic had an increased content of *Bifidobacterium* (*p* = 0.025), which was not observed with the placebo. However, the baseline and end-of-study liver fat contents did not change significantly in the prebiotic and placebo groups, neither did the liver function tests’ metabolic and inflammatory mediators, including fibroblast growth factor-19 and lipopolysaccharide-binding protein. Body weight remained stable in both groups. These findings suggest that prebiotic treatment without weight reduction is insufficient to improve NAFLD.

## 1. Introduction

Nonalcoholic fatty liver disease (NAFLD) is the most common liver disease in the Western world and is emerging as a major health problem globally. A recent meta-analysis estimated that 32% of the adult population is affected by NAFLD [1]. Fat accumulation in the liver, known as steatosis, can progress to inflammation, fibrosis, and, eventually, in a proportion of these individuals, to cirrhosis, liver transplantation, and hepatocellular carcinoma [2].

Numerous studies have shown a strong association between NAFLD and metabolic syndrome [3]. In fact, metabolic-dysfunction-associated steatotic liver disease (MASLD) has recently been suggested as the preferred terminology for fatty liver disease associated with metabolic syndrome [4]. Unfortunately, the only interventions with proven benefits in NAFLD are limited to lifestyle modifications, primarily weight loss and increased physical activity, to which adherence is often difficult. To date, there are no Food and Drug Administration (FDA)/European Medicines Agency (EMA)-approved pharmacological agents for the treatment of NAFLD [5].

Recent evidence suggests that an altered gut microbiota has a role in the development and progression of NAFLD [6,7]. Individuals with NAFLD show different gut microbiota signatures compared to healthy people [8,9,10]. One mechanism linking the disturbance of gut microbiota composition (known as dysbiosis) to liver damage is an increased intestinal permeability, which allows for the translocation of microbes, microbial products, and toxins into the liver (i.e., “leaky gut”) [7]. 

The translocation of dysbiotic bacteria can lead to metabolite production, which affects liver metabolism, such as via short-chain fatty acids (SCFAs), trimethylamine N-oxide (TMAO), bile acids (BAs), and lipopolysaccharide (LPS). SCFAs, which include acetate, propionate, and butyrate, act as sources of energy for the cells in the gut, strengthen the gut barrier, and maintain the optimal permeability of the gut, thereby preventing harmful substances from entering the bloodstream. SCFAs also possess anti-inflammatory properties and can improve insulin sensitivity, which are pivotal in the development and progression of NAFLD (for a review, see [11]). The intestinal microbiota promotes the conversion of choline into trimethylamine, which is subsequently metabolized into TMAO in the liver [12]. TMAO is a toxic compound that initiates inflammation in hepatocytes, promoting the advancement of NAFLD to nonalcoholic steatohepatitis (NASH) [13]. Increased production of TMAO reduces choline levels, resulting in a decrease in choline, consequently affecting the export of hepatic very-low-density lipoproteins and modulation of BA synthesis [12,14,15]. BAs are synthesized in the liver and are important signaling molecules. BAs activate the Farnesoid X receptor (FXR), which is currently considered to have a major role in NAFLD [16]. FXR regulates mucosal defense mechanisms, prevents bacterial overgrowth, and promotes epithelial integrity. FXR has also an important role in controlling hepatic triglyceride levels and inflammation. BA-induced effects that promote insulin sensitivity are mediated by fibroblast growth factor-19 (FGF-19). FGF19 is expressed in ileal enterocytes and released into enterohepatic circulation. Reduced levels of FGF-19 are observed in obesity and related disorders, including NAFLD [17,18]. Elevated LPS levels play a major role in the pathogenesis and progression of NAFLD by activating toll-like receptor 4 (TLR4) and the inflammatory cascade [19].

Prebiotics fibers are a group of nondigestible carbohydrates that modulate the human microbiota to a gut bacterial composition considered advantageous to the host’s health. Studies on animal models of NAFLD suggest that dietary supplementation with prebiotics fibers have a favorable effect on NAFLD by modifying gut microbiota, reducing body fat, and improving glucose metabolism. Furthermore, this may lead to improved gut-barrier function and reduced blood levels of the endotoxin LPS as measured by the more stable LPS-binding protein (LPS-BP) [20,21]. 

A meta-analysis including small randomized controlled trials conducted in patients with NAFLD found that microbial therapies (probiotics, prebiotics, and synbiotics—(PPS)) significantly reduced body mass index and alanine transaminase (ALT) levels but not markers of inflammation (tumor necrosis factor-alpha (TNF-α) and C-reactive protein (CRP)) [22]. However, these results fail to determine whether the decrease in liver enzymes is mediated by weight reduction or by a direct effect on gut microbiota.

Inulin-type fructans (ITFs) have been considered as high-quality prebiotics, which change the microbiome and promote the growth of *Bifidobacterium* [23,24]. A randomized double-blind trial comparing ITFs to a maltodextrin placebo in 30 obese women demonstrated improvements in glucose homeostasis and reduced levels of fecal SCFAs, which have a key role in host metabolism and immune responses [25,26]. A pilot randomized controlled trial (RCT) with a crossover design in which oligofructose or a placebo was administered for 8 weeks to seven patients with biopsy-proven NAFLD found a significant reduction in aspartate aminotransferase (AST) [27]. These studies support the potential of the use of ITFs in the management of NAFLD.

In order to evaluate the effect of prebiotics on NAFLD independent of weight reduction, we conducted an RCT of prebiotic supplementation in patients with NAFLD and metabolic syndrome while maintaining stable weight. The aims of the study were as follows: 1. to determine whether prebiotic supplementation affects liver fat content (LFC), as measured by H^1^MR spectroscopy (H^1^MRS), other liver-related parameters, metabolic profile, LPS-BP, and FGF-19 levels; 2. to determine whether prebiotic supplementation modifies the gut microbiota.

## 2. Materials and Methods

### 2.1. Trial Design

Patients were recruited at the Diabetes & Metabolic Institute and from the Gastroenterology and Hepatology Institute of Kaplan Medical Center. The trial was registered (www.cinicaltrials.gov, accessed on 25 November 2023, NCT02642172). The study protocol was approved by the Local Ethics Committee of Kaplan Medical Center (159-13-KMC). All participants provided written informed consent.

### 2.2. Eligibility Criteria

The inclusion criteria were as follows: age 18–70, diagnosis of NAFLD based on fatty infiltration detected by ultrasonography, and ALT ≥ 30 U/L [28]. Other inclusion criteria were overweight (BMI ≥ 27 kg/m^2^), fulfillment of the metabolic syndrome criteria according to the National Cholesterol Education Program [29], willing to participate in the study, and signing an informed consent form. The exclusion criteria included the following: evidence of other etiologies of chronic liver diseases, such as hepatitis B, hepatitis C, HIV, autoimmune diseases, and metabolic diseases, and evidence of cirrhosis, advanced liver disease, or hepatocellular carcinoma. We excluded individuals using medications with known hepatotoxicity, glucose-lowering drugs, probiotics, prebiotics, nonsteroidal anti-inflammatory drugs (NSAIDs), omega-3 fatty acids, vitamin E, and Silybum marianum in the 2 months preceding the study, as well as those who underwent recent treatment with antibiotics. Individuals who drank more than 20 g/day of alcohol for women and more than 30 g/day for men were excluded. Other exclusion criteria were as follows: presence of gastrointestinal or mental disorders, prior bariatric surgery, serious medical conditions, pregnancy, and consumption of unusual diets, e.g., vegetarian, vegan, ketogenic, or extreme hypocaloric diets.

### 2.3. Study Design

The study was a single-center, double-blind, and placebo-controlled intervention trial. Participants received either an 8 g prebiotic bid in the active group or a placebo supplement (i.e., maltodextrin) for 12 weeks. Study participants were blindly randomized to the active and placebo groups by the research staff. Two weeks before the randomization visit, participants came for a screening visit. At the screening visit, the following data were collected: demographics, medical history, lifestyle habits (smoking, alcohol consumption, diet, and physical activity). Participants were instructed to follow a weight-maintenance diet provided by a registered dietician and to maintain their usual physical activity.

The intervention consisted of 16 g per day of a prebiotic ITF (inulin/oligofructose 75/25) or placebo (maltodextrin) (both products were prepared by Hadassa Bymel-Pharmacy & Nature, Tirat Carmel, Israel, which purchased the inulin (Fibruline^®^) and the oligofructose (Fibrulose^®^ F97) from Cosucra Groupe, Warcoing, Belgium). The supplements had a similar appearance and taste as the prebiotic and were provided in unlabeled and identical opaque sachets. The powdered supplements were mixed into drinks, as chosen by the participants, and ingested (morning and evening). To allow for adaptation, the participants were instructed to consume one sachet per day in the first week and two sachets per day thereafter. For assessment of compliance, the participants were asked to return unused sachets.

Throughout the trial, the subjects underwent monthly visits to assess the adherence and ensure that weight was maintained. During these visits, the following data were collected: blood pressure and anthropometric measurements, bowel function, and gastrointestinal symptoms or side effects. In addition, phone calls were conducted every two weeks to ascertain compliance with the trial product and protocol.

Fasting blood serum samples were taken at baseline and at the end of the study for the following parameters: glucose, insulin, hemoglobin A1c (HbA1c), total cholesterol, high-density lipoproteins cholesterol (HDL-cholesterol), triglycerides, ALT, AST, γ-glutamyl transferase (GGT), CRP, FGF-19, and LPS-BP. In addition, fecal samples were collected for microbiota composition analysis. All participants underwent baseline and end-of-study H^1^MRS to evaluate the LFC. 

### 2.4. Measurements and Procedures 

Height and body weight were measured, and body mass index (BMI) was calculated. Waist circumference was measured by tape at the level of 0.5–1 cm above the umbilicus. Body fat percentage was assessed by bioelectrical impedance analysis [BF-508-Omron, Dalian, China]. 

Blood tests were performed using standardized methods at the Kaplan Medical Center Clinical Chemistry Laboratory. Low-density lipoprotein cholesterol (LDL-C) was calculated using the Friedewald equation. The Homeostasis Model Assessment of Insulin Resistance (HOMA-IR) was calculated using the following formula: fasting insulin (mU/L) × fasting glucose (mmol/L)/22.5. Serum LPS-BP was measured by ELISA (Human LBP ELISA Kit-Sigma-Aldrich, Saint Louis, MO, USA), and FGF-19 was determined by ELISA (R&D Systems, Minneapolis, MN, USA).

The LFC measurements were assessed by H^1^MRS using 3 Tesla Siemens Magnetom Prisma MRI Scanner (Erlangen, Germany). 

The fecal sample collection was conducted as follows: Feces were collected at home on the day before arriving to the clinical center, frozen immediately after defecation at −20 °C in the subject’s freezer, and transferred to the clinical center on ice. Samples were stored on arrival at −70 °C until analysis. 

The microbiota composition determination was performed as follows: DNA was extracted using the PowerSoil kit (MoBio, Carlsbad, CA, USA) according to the HMP (Human Microbiome Project) guidelines. PCR amplification of the 16S rRNA gene was carried out in a dedicated PCR cabinet with universal prokaryotic primers containing 5-end common sequences, as previously described [30] (CS1-341F 5’-ACACTGACGACATGGTTCTACANNNNCCTACGGGA-GGCAGCAG and CS2-806R 5’-TACGGTAGCAGAGACTTGGTCTGGACTACHVG-GGTWTCTAAT). Twenty-four PCR cycles (95 °C for 15 s, 53 °C for 15 s, and 72 °C for 15 s) were conducted using the PCR master mix KAPA2G Fast™ (KAPA Biosystems, Wilmington, MA, USA); successful amplification was verified by agarose gel electrophoresis. Products were shipped to the NGS center at HyLabs for the incorporation of Illumina adaptors and sample-specific barcodes in a 2nd 8-cycle PCR. Paired-end deep sequencing (2 × 250) of the PCR products was performed on an Illumina MiSeq platform [31]. A custom R script was used to identify and remove sequencing primers, and DADA2 [32] was then used for quality filtration (maxEE set at 2), inference of accurate sequence variants (ASVs), chimera removal, and taxonomic assignment against the Silva database v138. To reduce sequencing bias, data were rarefied to an equal depth of 3000 seqs/sample. Differentially abundant taxa were detected using the LEfSe [33] biomarker detection tool; R package vegan-2.6.4 was used to calculate the Shannon diversity index and fit the permutational analysis of variance (PERMANOVA) models. 

### 2.5. Statistics 

Continuous variables are represented as medians and interquartile ranges (25% to 75%). Mann–Whitney U tests were used to compare continuous variables to test for differences between the placebo and prebiotic groups. Categorical variables are presented as frequencies. Differences among categorical variables were analyzed by Fisher’s exact test. Wilcoxon signed-ranks tests were conducted to examine the differences between baseline and 12 weeks of treatment in the placebo and prebiotic groups. A p-value of 0.05 is considered statistically significant. The data in the graphs are represented by box-and-whisker plots, The bold line represents median. The statistical analysis was performed using SPSS software version 26 (SPSS Inc., Armonk, NY, USA).

## 3. Results 

### 3.1. Baseline Characteristics

Twenty-four subjects were screened, two subjects declined to participate after the screening visit and twenty-two participants were included and blindly assigned to two groups receiving the prebiotic or placebo. Two participants withdrew from the study at weeks 6 and 8 due to work commitments that conflicted with the study’s requirements, and one was excluded because of the use of antibiotics (for pharyngitis). Thus, a total of 19 patients with NAFLD (11 in the placebo group and 8 in the prebiotic group) completed the study with good compliance throughout the study’s duration. None of the patients reported any discomfort, side effects, or symptoms associated with the treatment. 

The baseline characteristics of the two treatment groups are summarized in Table 1. At baseline, there were no significant differences in the demographic, anthropometric, clinical, and biochemical parameters. Furthermore, there was no statistically significant difference in the baseline MRS-measured LFCs. 

**Figure 1:**
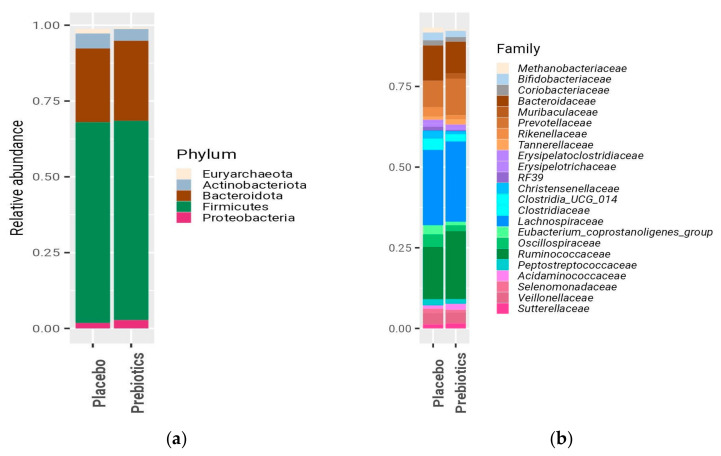
Microbial composition at baseline across the treatment groups. Mean relative abundances (RAs) are shown at the (**a**) phylum level and (**b**) family level. Taxa are color coded according to the following phylogenetic lineages: beige for Euryarcheota; gray for Actinobacteriota; brown for Bacteroidota; and violet for Proteobacteria. The Firmicutes phylum, because of its diversity, was further coded at the class/order level, as follows: families belonging to Bacilli are shown in purple; *Veillonellales–Selenomonadales* in pink; *Oscillospirales* in green; *Lachnospirales* in blue; and other Clostridia in cyan. Only taxa with a mean RA of 0.005 or higher (phylum level), of 0.01 or higher (family level) are shown.

The microbial composition at baseline (Figure 1), as expected for human gut microbiome, was dominated by members of the Firmicutes phylum [mean relative abundances (RAs) of 65% and 66% in the prebiotic and placebo groups, respectively] and the Bacteroidota phylum (26.5%/24% in prebiotic/placebo). The microbial compositions did not significantly differ between the two groups at baseline; similar alpha diversity indices were obtained for both groups (Shannon index: 3.13 vs. 3.19, n.s.), and no significant association between microbiome and study group was observed from the permutational analysis of variance (PERMANOVA). There was also no significant difference in the *Bifidobacterium* relative abundances (mean RAs in the prebiotic/placebo groups of 0.016 vs. 0.019, respectively; *p* = 0.4, Figure 1b).

### 3.2. Anthropometrics Measurements, Bifidobacterium, LFC, and Biochemical Blood Test

**Figure 2:**
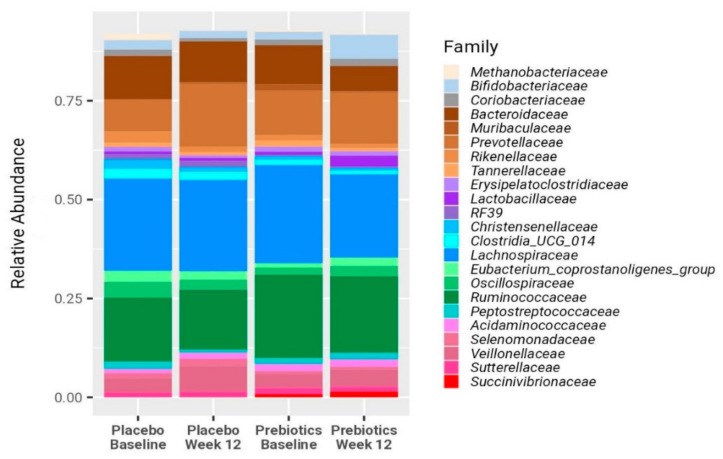
Microbial compositions at baseline and at 12 weeks for the prebiotic and placebo groups. Bars represent the mean relative abundances of the microbial families and are color coded according to the following phylogenetic lineages: gray for families belonging to the Actinobacteria class; brown for Bacteroidia; purple for Bacilli; and pink for *Veillonellales–Selenomonadales*. Firmicutes are subdivided into green (for *Oscillospirales*), blue (*Lachnospirales*), and cyan (other Clostridia). Only families with a mean RA of 0.01 or higher are shown.

**Figure 3:**
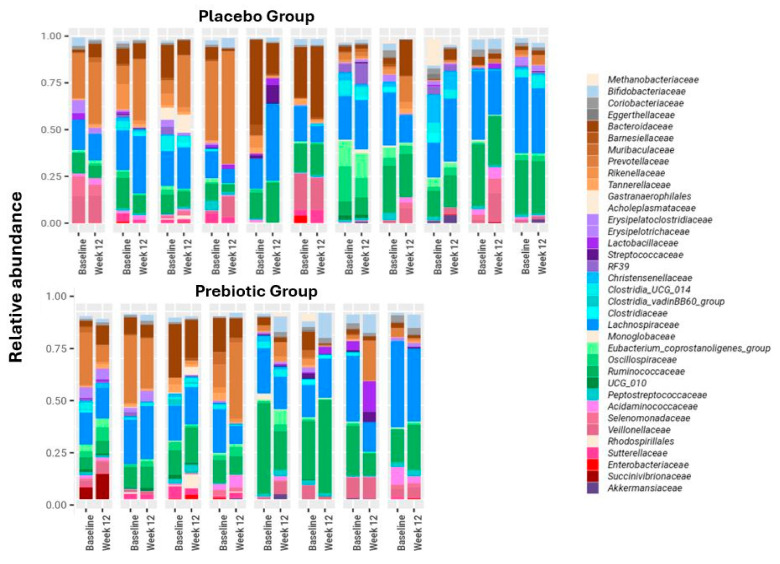
Microbial composition at baseline and at 12 weeks per patient. The microbial composition is highly personalized and temporally stable. The relative abundances of the microbial families are shown per patient, at baseline, and at 12 weeks. Each panel represents one patient. The color coding of the families represents the bacterial lineage, as in Figure 1 and Figure 2. Across all samples only families with an RA of 0.02 or higher are shown.

**Figure 4:**
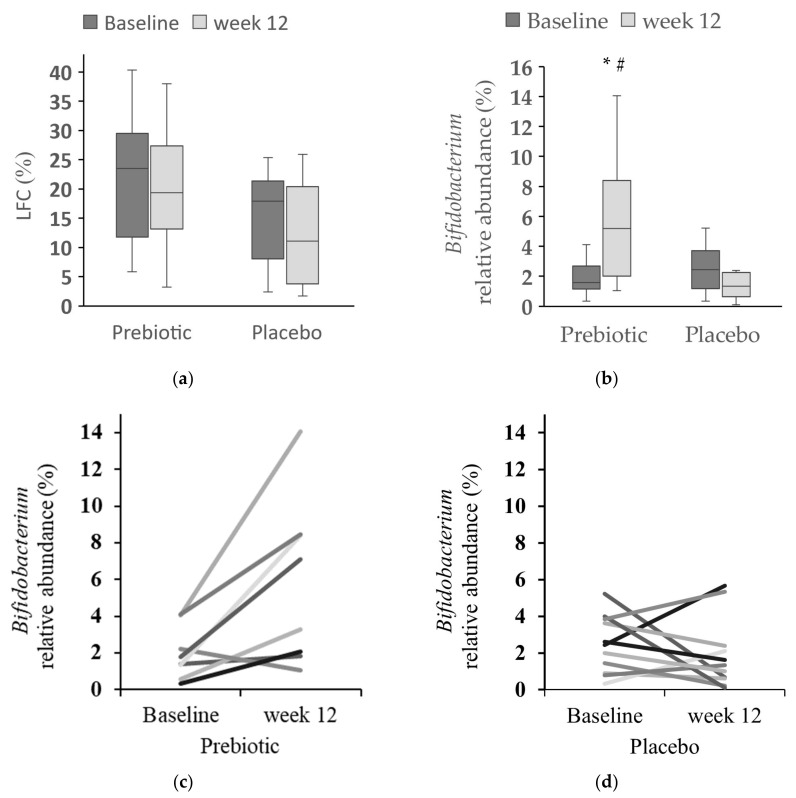
*Bifidobacterium* and LFC in both treatment groups. Variables in (**a,b**) are represented by box-and-whisker plots. The bold lines represent medians. (**a**) LFC; (**b**) relative abundance of *Bifidobacterium*; (**c**) subject-specific response of the *Bifidobacterium* relative abundance before and after the prebiotic treatment; (**d**) subject-specific response of *Bifidobacterium* before and after the placebo treatment. * Prebiotic group at week 12 vs. baseline, *p* = 0.025; # Week-12 prebiotic group vs. placebo group, *p* = 0.02. LFC = liver fat content. Different shades of gray were used for better visibility and distinction of individual trends for each participant.

BMI, body weight, body fat percentage, and waist circumference remained stable in both groups (Table 2). Likewise, microbial composition remained overall stable for all participants (Figure 2 and Figure 3). After 12 weeks no significant difference in Shannon diversity index was found between the two treatment groups or between baseline and post-treatment. Furthermore, a PERMANOVA model, built to explore associations between microbiome and patient identity, time point, and treatment group, identified only patient identity as a factor significantly associated with the microbial composition; as expected for longitudinal cohorts, 76% of the variance in the microbiome was explained by this factor (*p* = 0.001; Figure 3). However, the application of the biomarker discovery algorithm Linear Discriminant Analysis Effect Size (LEfSe) identified *Bifidobacterium* as the only bacterial genus that increased significantly (LDA = 4.3, *p* = 0.02) in the prebiotic, but not in the placebo group (Figure 2, Figure 3 and Figure 4b–d). Twelve weeks of treatment with prebiotic led to a 3.2-fold increase in the mean *Bifidobacterium* relative abundance, 0.052 (0.019–0.084) after treatment vs. 0.016 (0.009–0.031) at baseline, *p* = 0.025. There was no significant change in the *Bifidobacterium* abundance in the placebo group (*p* = 0.53). At the end of the study, the *Bifidobacterium* abundance was 4-fold higher on average in the prebiotic group compared with the placebo, 0.052 (0.019–0.084) vs. 0.013 (0.006–0.024), *p* = 0.02 (Figure 4b). However, the change in the bacterial composition in the prebiotic group did not result in a significant change in the H^1^MRS-measured LFCs, (baseline: 24% (12–30); vs. 12 weeks: 19% (13–27), *p* = 0.3) (Figure 4a). Likewise, there were no significant changes in the liver function tests, fasting lipid profiles, fasting plasma glucose, insulin, HbA1c, HOMA-IR, and CRP levels following the treatment in both groups (Table 2)**.**

### 3.3. LPS-BP and FGF-19

**Figure 5:**
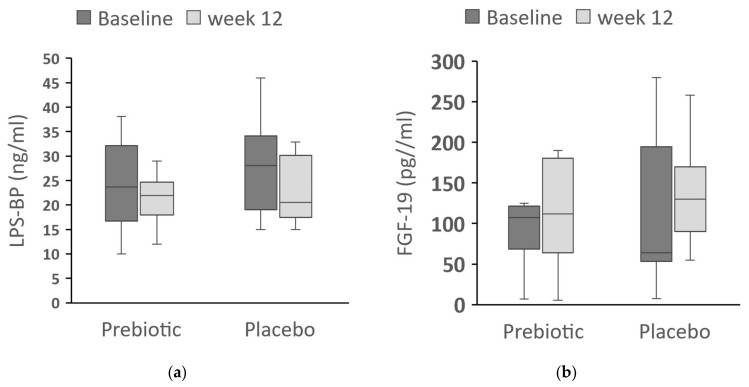
FGF-19 and LPS-BP in both of the treatment groups. Variables in (**a**,**b**) are represented by the box-and-whisker plots. The bold lines represent medians. LPS-BP (**a**) and FGF-19 (**b**) at baseline and at week 12 of prebiotic or placebo intake, (both *p* > 0.05). LPS-BP = lipopolysaccharide-binding protein; FGF-19 = fibroblast growth factor-19.

In order to determine whether the prebiotic treatment affected the metabolic endotoxemia LPS-BP was measured and we did not find any significant changes between the baseline and 12 weeks in both groups. LBP in the prebiotics: 24 (10–32) ng/mL vs. 22 (17–25) ng/mL (*p* = 0.2); placebo: 28 (18–39) ng/mL vs. 20 (17–30) ng/mL (*p* = 0.2) (Figure 5a). 

We also measured FGF-19, which has been shown in animal studies to have beneficial effects on glucose homeostasis and on hepatic steatosis. However, we did not find significant differences between the baseline and week-12 levels in either the prebiotic, 108 (64–123) pg/mL vs. 112 (62–184) pg/mL (*p* = 0.3), or in the placebo group, 64 (47–220) pg/mL vs. 130 (75–175) pg/mL (*p* = 0.9) (Figure 5b).

## 4. Discussion

In this current randomized controlled pilot study conducted on patients with NAFLD, we found that in the absence of weight loss, 12 weeks of prebiotic treatment (ITF supplementation) markedly increased the abundance of fecal *Bifidobacterium* species with no significant changes in LFC or other liver-related parameters, including metabolic endotoxemia and FGF-19 levels. 

ITFs were selected as the prebiotic in our study, since this mixture of inulin and oligofructose was shown previously to have an optimal effect on gut microbiome with a strong bifidogenic effect (approximately 1.8–3.8 times higher). Other impacts on fecal microbiota composition varied more widely, such as an increase in *Lactobacillus* and *Faecalibacterium prausnitzii.* It has been suggested that these alterations in the intestinal microbiota composition may be beneficial to human health, as follows: improvement in intestinal barrier function, improvement in laxation, increase in insulin sensitivity, improvement in lipid profile, increases in the absorption of calcium and magnesium, and increase in satiety [34]. 

Recently, ITFs have been shown to significantly increase levels of *Bifidobacterium* in obese adult patients while reducing calprotectin, a marker for intestinal inflammation [35]. Similarly, children who were overweight or obese and given oligofructose-enriched inulin showed increased abundance of *Bifidobacterium* and a reduction in the pro-inflammatory cytokine interleukin-6 in their blood [36]. Since patients with NAFLD are generally overweight or obese, supplementing their diet with *Bifidobacterium*-promoting fiber was, therefore, a therapeutic approach worth exploring.

Our study results do not support previous animal models in which PPS supplementation aimed at altering gut microbiota in NAFLD has shown promising outcomes [37,38,39,40,41,42,43,44,45,46]. In these models, PPS demonstrated beneficial effects of restoring intestinal microbiota composition, improving intestinal barrier integrity, and reducing endotoxin levels [47]. A recently published study in rats showed that inulin treatment attenuated hepatic steatosis via the modulation of gut microbiota composition, maintaining the intestinal barrier function. Inulin administration significantly enhanced *Bifidobacterium*, *Phascolarctobacterium,* and *Blautia*; conversely, opportunistic pathogens, such as *Acinetobacter* and *Corynebacterium_1*, were suppressed [46]. In our study, in the prebiotic group, only an increase in *Bifidobacterium* was observed, with no significant difference in the Shannon diversity index. Therefore, the significant effect on attenuating hepatic steatosis observed in this rat study may be attributed to prebiotics eliciting greater modifications in the gut microbiota compared to humans.

Recently, there is a growing interest in other prebiotics such as pectin, which has been shown in animal models to induce not only an increase in *Bifidobacterium* but also *Bactereroides*, *Lactobacillus,* and *Lachnospiraceae* abundances (for Review Hu et al. [48]). Pectin in those animal models was found to have the beneficial effects of inducing an increased expression of genes involved in fatty acid oxidation and decreased expression of genes involved in lipogenesis. This led to an improvement in the hepatic steatosis and decreases in ALT and inflammatory cytokine levels [47,48]. However, the usage of pectin led to a reduced caloric intake, which could partially contribute to these beneficial effects [48]. Other possible limitations in humans may be due to the poor palatability of large amounts of pectin and the associated side effects, such as increased abdominal discomfort [47].

Proposed mechanisms for the beneficial effects of prebiotics include the modification of metabolites, such as SCFAs, branched-chain amino acids (BCAAs), and BAs [6,43]. In addition to their function in lipid absorption, BAs are signaling molecules that are believed to play a pivotal role in NAFLD via the activation of FXR and Takeda-G-protein-receptor-5 (TGR5). FXR regulates mucosal defense mechanisms by promoting epithelial integrity and by preventing bacterial overgrowth. FXR is also important in controlling hepatic triglyceride accumulation, increasing insulin sensitivity, and improving glucose homeostasis. TGR5 has a role in inhibiting inflammation [6,12]. 

In contrast to the beneficial effects of PPS observed in animal models, human studies have yielded conflicting findings. These inconsistencies can be attributed to variations in study methodologies and PPS products [49], as well as baseline intestinal microbiota composition [34]. Two pilot human studies previously investigated the effect of oligofructose in NASH patients. In a placebo-controlled, randomized trial, individuals with NASH were randomized to receive prebiotic oligofructose (*n* = 8) or isocaloric placebo (*n* = 6) for 9 months. The supplementation of prebiotic supplementation resulted in an increased abundance of *Bifidobacterium* and improvement in liver histology. The study was small but was longer in duration compared to our study. As in our study, the patients neither lost weight nor exhibited improvements in serum ALT and GGT concentrations or improvements in glycemic control, serum insulin levels, HOMA-IR, and LPS [50]. In an 8-week crossover RCT with oligofructose or placebo in seven individuals with biopsy-proven NAFLD, FOS led to a minor yet significant reduction in AST but did not lead to a significant change in ultrasound-measured steatosis [27].

A meta-analysis including 1309 patients from 25 different studies analyzed different PPS in patients with NAFLD. In nine studies (16.5% of the combined study populations), prebiotics were used [22]. All types of PPS resulted in a reduction in BMI, hepatic enzymes (ALT, AST, and GGT), and improved lipid profile but had no significant effect on the biomarkers of inflammation, TNF-α, and CRP. Another meta-analysis that analyzed the effect of different PPS also found a favorable metabolic effect but there was no reference to the effect on weight [51]. These studies have several limitations. First, liver status was assessed only by measurement of liver enzymes without additional parameters, such as LFC. Second, the microbiome alterations consequent to PPS interventions were not reviewed, and, thus, it is not possible to ascertain whether the PPS played a role in the observed improvements. Finally, some of the metabolic and liver effects of PPS can be attributed to the effect of weight reduction alone [52]. 

Similar to our study, recent investigations into NAFLD treatment with PPS have aimed to preserve participants’ lifestyle habits. A pilot study was conducted in patients with NAFLD administered probiotics for six months without lifestyle-modifying intervention. This study did not result in any significant clinical improvements in hepatic steatosis, fibrosis, and activity score, as well as in the biochemical blood tests. Notably, the absence of gut microbiota analysis prevents the assessment of any potential changes in the microbiome following probiotic supplementation [53]. In another double-blind, placebo-controlled clinical trial treating NASH patients with probiotic supplementation for 6 months led to a reduction in the AST-to-platelet ratio index but failed to improve liver steatosis, fibrosis, and inflammatory activity. Of note, in this study, probiotic supplementation did not change gut microbiota composition or anthropometric indices [54]. Therefore, the lack of improvement in these studies may be attributed to the probiotic preparation’s ineffectiveness on the microbiome.

In the notable relatively large randomized INSYTE trial that included 104 NAFLD patients, synbiotic administration over 10–14 months induced alterations in fecal microbiomes, increasing the abundance of *Bifidobacterium* and *Faecalibacterium*. However, these changes did not reduce LFC or markers of liver fibrosis. Interestingly, multivariate analysis revealed that 1 kg weight loss was independently associated with reduced LFC and improved fibrosis scores [55].

In our current study, the participants’ weights were maintained by a weight-maintenance diet with a strict follow-up by a dietitian to prevent the potential confounding effect of weight loss. Furthermore, we also noted an elevation in *bifidobacterium* levels following prebiotic administration. However, this increase did not correspond to any significant changes in the parameters under investigation.

Taken together, these results imply that avoiding weight reduction seems to abolish the beneficial effects seen in some previous studies on the effects of prebiotics in patients with NAFLD. This implies that while prebiotic supplementation may positively influence fecal microbiota composition, its impact on liver fat accumulation and metabolic parameters in patients with NAFLD may be limited. 

It has been shown in animal models that probiotics and prebiotics supplementation can ameliorate NAFLD mediated by their favorable effect on gut dysbiosis, improve gut barrier function, and, subsequently, lead to reductions in pro-inflammatory cytokine and LPS levels [37,56,57]. LPS levels play a major role in the pathogenesis and progression of NAFLD by activating TLR4 and the inflammatory cascade [19]. 

Recognizing the pivotal role of LPS in NAFLD pathology, we included an assessment of LPS levels in our study to elucidate the effects of prebiotic intervention on this key inflammatory marker. However, in our study, prebiotic ITF treatment did not significantly alter LPS-BP levels. Previous human studies have provided conflicting findings. Two studies using synbiotics/prebiotics without significant weight loss failed to show any effect on LPS blood levels [50,55,58]. In another study combining multistrain probiotic treatment with lifestyle modification led to an improvement in the pro-inflammatory cytokine profile compared to the placebo group [59]. It is worth noting the uniqueness of our study, wherein we employed the more stable LPS-BP as a measure, providing potentially more accurate information. In addition, we instructed patients not to alter lifestyle habits. This may suggest a potential interplay between lifestyles, microbiota modulation, and LPS levels in the context of NAFLD management, warranting further exploration.

In recent years there has been a growing interest in the family of endocrine gut-derived FGFs with a special interest in their role in NAFLD/NASH. FGF-19 is expressed in the ileal enterocytes in response to BA stimuli. Fasted serum FGF-19 levels are reduced in individuals with overweight, obesity, and NAFLD and it has even been suggested as a diagnostic biomarker for NASH [17]. Since gut microbiota has a profound effect on BA metabolism [60], we were interested in determining whether prebiotic supplementation affected FGF-19 levels. In our study, prebiotic treatment did not affect circulating levels of FGF-19. To the best of our knowledge, no previously published study has evaluated the effect of PPS on FGF-19 levels in patients with NAFLD. 

FGF-21, which also belongs to the FGF family, is highly expressed in the liver and is released in response to metabolic stimuli such as high glucose and free fatty acids. In various NASH animal models, FGF-19/21 analogues decreased steatosis, inflammation, and fibrosis [18]. Consequently, FGF-19/21 analogues are now being considered as a potential treatment for NASH. For instance, in a phase 2 study involving patients with NASH, 12 weeks of Aldafermin (FGF-19 analogues) reduced absolute liver fat, as measured by magnetic resonance imaging proton density fat fraction (MRI-PDFF), by 5% in 80% of the patients. Additionally, a significant decrease in plasma liver enzymes ALT and AST was observed. Similarly, a phase 2b trial demonstrated that treatment with the FGF-21 analogues pegozafermin, administered at doses of 30 mg once weekly and 44 mg every 2 weeks for 24 weeks, led to significant improvements in fibrosis compared with placebo [61]. Pegozafermin showed an acceptable safety profile and efficacy [61,62]. Our preliminary results may imply that further trials directed at the manipulation of gut microbiota should take into consideration the effects on both FGF-19 and FGF-21. 

Our study has several strengths, including the assessment of gut microbiota composition before and after treatment, as well as the use of accurate noninvasive techniques, such as proton H^1^MRS to measure LFC [63]. Moreover, we evaluated not only liver, metabolic, and inflammatory parameters but also measured FGF-19 levels, which is believed to play a pivotal role in the pathogenesis of NAFLD/NASH and a promising treatment modality. We also carefully excluded patients who took dietary supplements or medications with potential hepatoxicity or that are known to modify the microbiome (e.g., metformin). Finally, our patients were instructed to maintain their regular lifestyle habits during the study period and were followed and monitored throughout the study to ascertain whether a stable weight was maintained. 

The study’s limitations are as follows: The main limitation of the study is the small sample size due to the strict inclusion and exclusion criteria and to the difficulty in recruiting participants during the COVID-19 pandemic. Therefore, this is a pilot study with limited statistical power. In addition, the follow-up period may have been too short to detect significant changes. We also did not evaluate other prebiotics such as pectin that may lead to more diverse effects on the microbiome. 

## 5. Conclusions

In this pilot randomized controlled trial, we observed that although prebiotic treatment led to a significant increase in fecal *Bifidobacterium* relative abundance, this did not result in a favorable effect on LFC and other hepatic, metabolic, and inflammatory parameters or in significant changes in FGF-19 levels. Our results concur with a few previous studies emphasizing the notion that PPS supplementation without weight loss is not sufficient for improvement in liver and metabolic measures in patients with NAFLD. 

## Figures and Tables

**Table 1:** Baseline characteristics of the study participants: demographic, anthropometric, clinical, metabolic, and liver parameters.

	Placebo (*n* = 11)	Prebiotic (*n* = 8)	*p*
Age (years)	50.0 (40.9–60.5)	47.8 (44.4–58.4)	1.0
Gender, male/female	9/2	6/2	0.7
Hypertension			
Yes	4	3	0.9
No	7	5	
Prediabetes			
Yes	5	4	0.8
No	6	4	
Diabetes			
Yes	2	0	0.2
No	9	8	
Anthropometrics			
Weight (kg)	94.4 (85.9–98.4)	94.1 (85.7–103.0)	0.7
BMI (kg/m^2^)	32.5 (30.2–35.1)	32.6 (29.5–33.2)	0.7
Waist circumference (cm)	107 (99–112)	106 (95–110)	0.6
Body fat (%)	34 (29–35)	33 (30–41)	0.9
Blood Pressure			
SBP (mmHg)	123 (120–128)	125 (117–132)	0.8
DBP (mmHg)	81 (76–83)	79 (77–82)	1
Blood chemistries			
Fasting glucose (mg/dL)	100 (90–110)	100 (93–105)	0.8
Insulin (IU/dL)	11.6 (9.0–20.8)	13.7 (7.6–21.4)	0.9
HbA1c (%)	5.8 (5.5–5.9)	5.6 (5.4–5.8)	0.1
HOMA-IR	3.7 (2.1–5.1)	3.5 (1.9–5.5)	0.9
Total cholesterol (mg/dL)	189 (145–228)	197 (181–210)	0.5
HDL-C (mg/dL)	41 (32–42)	38 (34–44)	0.6
LDL-C (mg/dL)	113 (94–150)	113 (87–141)	0.7
Triglycerides (mg/dL)	197 (125–296)	168 (138–259)	0.8
ALT (IU/L)	50 (30–69)	36 (31–54)	0.6
AST (IU/L)	25 (20–39)	27 (25–29)	0.5
GGT (IU/L)	40 (28–102)	34 (27–48)	0.6
CRP (mg/dL)	2.5 (1.8–5.7)	3.0 (1.3–5.0)	1
LFC (%)	18 (8–21)	24 (12–30)	0.2

Continuous variables are presented as medians and interquartile range (25% to 75%). Categorical variables are presented as frequencies. BMI = body mass index; SBP = systolic blood pressure; DBP = diastolic blood pressure; HbA1c = hemoglobin A1c; HOMA-IR = Homeostasis Model Assessment of Insulin Resistance; HDL-C = high-density lipoprotein cholesterol; LDL-C = low-density lipoprotein cholesterol; ALT = alanine aminotransferase; AST = aspartate aminotransferase; GGT = gamma-glutamyl transferase; CRP = C-reactive protein; LFC = liver fat content.

**Table 2:** Baseline and week-12 characteristics of the study participants: anthropometric, clinical, metabolic, and liver parameters.

	Placebo (*n* = 11)			Prebiotic (*n* = 8)		
	Baseline	Week 12	*p*-Value	Baseline	Week 12	*p*-Value
Weight (kg)	94.4 (85.9–98.4)	91.5 (83.5–98.9)	0.5	94.1 (85.7–103.0)	93.9 (85.0–100.9)	0.1
BMI (kg/m^2^)	32.5 (30.2–35.1)	31.4 (29.7–36.0)	0.5	32.6 (29.5–33.2)	32.4 (29.7–32.9)	0.3
Waist circumference (cm)	107 (99–112)	106 (95–112)	0.1	106 (95–110)	106 (93–110)	0.3
Body fat (%)	34 (29–35)	32 (29–41)	0.3	33 (30–41)	34 (28–40)	0.2
Fasting glucose (mg/dL)	100 (90–110)	96 (88–110)	0.7	100 (93–105)	101 (95–106)	0.6
Insulin (mU/L)	11.6 (9.0–20.8)	10.4 (7.4–14.7)	0.3	13.7 (7.6–21.4)	14.7 (9.8–19.9)	0.6
HbA1c (%)	5.8 (5.5–5.9)	5.7 (5.5–6.0)	0.9	5.6 (5.4–5.8)	5.6 (5.4–5.8)	0.2
HOMA-IR	3.7 (2.1–5.1)	2.82 (1.6–3.7)	0.2	3.5 (1.9–5.5)	3.7 (2.3–5.1)	0.7
Total Cholesterol (mg/dL)	189 (145–228)	199 (148–239)	0.7	197 (181–210)	199 (178–236)	0.1
HDL-C (mg/dL)	41 (32–42)	35 (30–42)	0.5	38 (34–44)	38 (35–41)	0.7
LDL-C (mg/dL)	113 (94–150)	127 (90–154)	0.7	113 (87–141)	103 (91–134)	0.6
Triglycerides (mg/dL)	197 (125–296)	206 (152–252)	0.6	168 (138–259)	176 (150–272)	0.7
ALT (U/L)	50 (30–69)	44 (30–52)	0.2	36 (31–54)	42 (28–50)	0.5
AST (U/L)	25 (20–39)	22 (21–30)	0.27	27 (25–29)	25 (22–41)	0.5
GGT (U/L)	40 (28–102)	32 (29–83)	0.3	34 (27–48)	40 (26–50)	0.9
CRP (mg/dL)	2.5 (1.8–5.7)	1.7 (1.1–4.5)	0.1	3.0 (1.3–5.0)	3.0 (1.1–4.4)	0.3
LFC (%)	18 (8–21)	11 (4–20)	0.4	24 (12–30)	19 (13–27)	0.3

Continuous variables are presented as medians and interquartile ranges (25% to 75%). Categorical variables are presented as frequencies. BMI = body mass index; HbA1c = hemoglobin A1c; HOMA-IR = Homeostasis Model Assessment of Insulin Resistance; HDL-C = high-density lipoprotein cholesterol; LDL-C = low-density lipoprotein cholesterol; ALT = alanine aminotransferase; AST = aspartate aminotransferase; GGT = gamma-glutamyl transferase; CRP = C-reactive protein; LFC = liver fat content.

## Data Availability

Raw 16S data and all clinical metadata have been deposited in [https://www.ncbi.nlm.nih.gov/sra/PRJNA1100376] NCBI’s Sequence Read Archive (SRA), Project ID PRJNA1100376.
